# A Comparative Analysis of Printed Circuit Boards with Surface-Mounted and Embedded Components under Natural and Forced Convection

**DOI:** 10.3390/mi13040634

**Published:** 2022-04-17

**Authors:** Maksim Korobkov, Fedor Vasilyev, Vladimir Mozharov

**Affiliations:** 1 Department of Digital Technologies and Information Systems, Moscow Aviation Institute (National Research University), 125993 Moscow, Russia; josef_turok@bk.ru; 2 Kiratech srl, 37135 Verona, Italy; v.a.mozharov@gmail.com

**Keywords:** PCB reliability, embedded components, finite element analysis, thermal analysis

## Abstract

This article is dedicated to the research of the physical reliability of electronic devices. It consists of a comparative thermal analysis of the cooling efficiency of a surface-mounted and an embedded component on a printed circuit board. A simulated finite element model of heat distribution over a printed circuit board with a surface component was constructed. An experiment confirmed the objectivity of the modeling results. The component’s temperature was then analyzed depending on the installation method (surface and embedded) and the cooling method (natural and forced with varying airflow velocities). The results showed that the temperature of the embedded component was less than the temperature of the surface-mounted component under natural convection and, in most cases, under forced convection (with an airflow velocity of forced cooling under $16\;\frac{m}{s}$).

## 1. Introduction

The embedding of electronic components into the base of a printed circuit board (PCB) is a promising direction for developing modern electronic system components for applications such as measuring, navigation [1,2,3], and electric power systems [4,5]. Furthermore, this technology potentially reduces the size of manufactured electronic devices, increases the density of interconnections, allows the application of new design solutions, improves device performance by placing components closer together and reduces parasitic inductances and capacitances [6,7]. In addition, the use of embedded components should increase the manufacturability of devices because the production of such PCBs combines the manufacturing and assembly processes.

However, the issue of ensuring the reliability of both electronic devices, in general, [8,9,10,11,12,13,14,15,16,17,18] and PCBs with embedded components, in particular [19,20], is very relevant. It is especially necessary to research the processes that led to breakage (failure physics), and the development of design rules based on these processes to ensure the reliability of electronic devices (design for reliability) [21]. For example, it has already been experimentally proven that, under thermal cycling [22] and drop test conditions [23], PCBs with embedded components are more reliable than PCBs with surface-mounted components.

**Figure 1:**
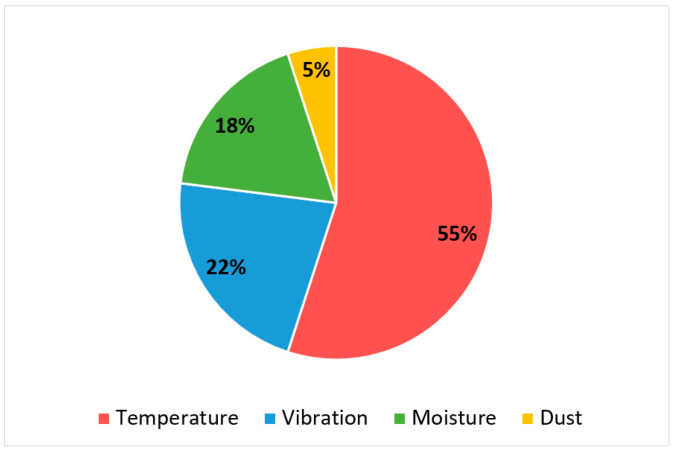
Distribution of the most frequent causes of electronic failures [22].

Below (Figure 1) is the distribution between the physical factors that most often cause failures in electronic modules [24,25,26]. Temperature fluctuations and vibrations are the main causes of deformation in PCBs and the connections on it. This leads to a breakdown in the electrical contact, detachment of the component, delamination of the base material and the appearance of cracks in it, as well as the destruction of metalized holes. Humidity and dust cause contamination, leading to the destruction of conductors and elements. In addition, humidity induces electrochemical migration processes: it causes short circuits between conductors and leads to the formation of conductive anodic filaments (CAF).

More than half of failures are due to thermal influences due to the wide variety of materials that make up the electronic device. They have different characteristics that should agree with each other. Below are some reasons for thermal failures in electronic modules:

1. the difference between the thermal expansion coefficients of the base material, electronic components, and solder leads to the destruction of solder joints;
2. the difference between the coefficients of thermal expansion of the base material and the material of the conductors leads to the destruction of metalized transition holes;
3. exposure to high temperatures, including heating of components in the absence of the required cooling system, can lead to PCB’s deformations of various kinds, such as warping and twisting and its stratification.

Solving these problems is necessary, especially for power electronics. In addition, the use of embedded components can be used to increase temperature stability and improve the cooling efficiency of electronics. Embedding components inside PCBs allow, on the one hand, the provision of better heat transfer directly from the component but, on the other hand, also reduce the heat transferred by air cooling. Based on the above, a comparative analysis of thermal processes in PCBs with embedded components and PCBs with a traditional surface mounting method is of interest.

## 2. Materials and Methods

The experimental study of thermal processes is associated with some difficulties, including significant costs associated with the production of samples, the definition of test methods and the variation of parameters between experimental samples. Simulation modeling based on finite element analysis (FEA) facilitates such a study. However, very often, incorrect models are used.

Therefore, for thermal analysis the following were required:

1. The execution of an experiment to determine the heat distribution on the PCB with a surface component;
2. The construction of a simulation finite element model is constructed, the correctness of which is confirmed by the results of the experiment;
3. The expansion of a simulation model, and the execution of a comparative analysis of thermal processes in PCBs with embedded components and PCBs with a surface mounting method.

### 2.1. Experiment to Determine the Thermal Distribution on a PCB

The most straightforward heat source is a resistor attached to an FR-4 fiberglass work piece. By applying a voltage to the resistor terminals, we obtained a picture of the heat distribution on the work piece using a thermal imager.

**Figure 2:**
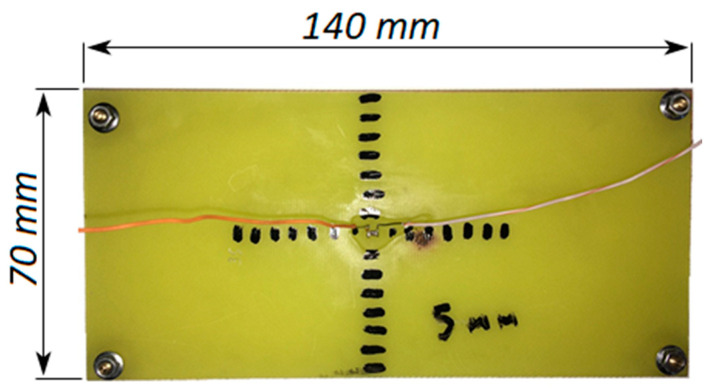
Experimental model.

A resistor was fixed with glue on a 1.5 mm thick FR-4 fiberglass billet in the 0805 case with 510±1% Ohm resistance. When gluing, the maximum contact of the resistor with the fiberglass was ensured. Wires for connecting electric current were soldered to the side surfaces of the resistor. It should be noted that the wires soldered to the resistor were a strong heat sink. Thus, the diameter of the wires should be chosen as the minimum necessary. In our case, we wanted to ensure the heat dissipation of the resistor from 0.5 to 1 W, and we applied a voltage of 20 V to the resistor terminals. Then, the power allocated was 0.784 W, and the current was approximately 39 mA. Considering the short duration of exposure and the experimental nature of use, we used AWG 24 wires with a diameter of 0.51 mm to supply current. To further simplify the analysis of the heat distribution over the PCB, risks were applied to the board in 5 mm increments. The marks were needed to analyze the image obtained using a thermal imager. The created experimental model is shown in Figure 2.

**Figure 3:**
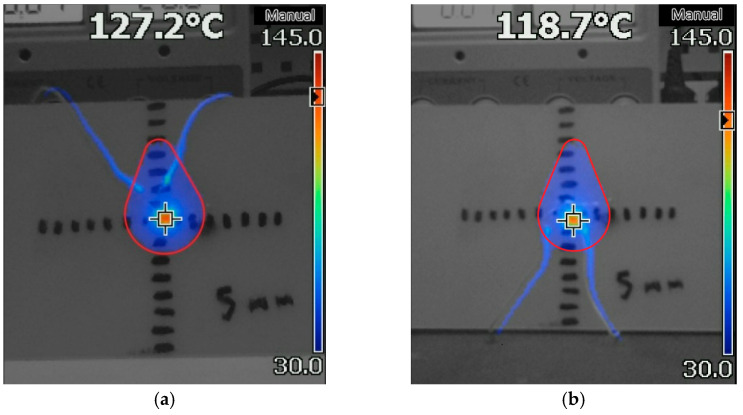
The picture of the heat distribution on the work piece during the experiment; the red contour highlights the heating area in which the temperature was above 303 K: (**a**) wires were laid over the work piece; (**b**) wires were laid under the work piece.

The experiment involved heating the work piece in a vertical position. Below (Figure 3) is a picture of the heat distribution obtained during the experiment (the dissipated power was 0.784 W).

As can be seen, in the vertical position, a drop-shaped pattern was observed, which showed the fact of convection heating of the work piece over the component. According to the results of a series of measurements, the steady-state temperature was 405±2 K. In addition, the arrangement of wires on the work piece did not affect the pattern of heat distribution: the results of the experiment with different arrangements of wires were almost identical (Figure 3).

Based on the obtained picture of the distribution of heat and temperature of the component, a future assessment of the correctness of the constructed simulation model was possible.

### 2.2. Development of a Simulation Model of Thermal Processes

To objectively model conduction and convection processes of heat transfer, we needed to choose software that allowed computational fluid dynamics (CFD) problems to be solved. Therefore, to solve this problem, the “Fluid Flow” module of the ANSYS software package was selected using the Fluent solver.

Also, several additional conditions needed to be imposed on the simulation model, taking into account all the particular features of the model, which are called boundary conditions [27]. The boundary conditions included the geometric model of the system, the physical properties of the system and the materials it consists of and the parameters of the process.

As a geometric model, a 3D model of the work piece was created, consisting of a 50×50×1.5 mm FR-4 fiberglass base, with a simplified model of a 2×1.2×0.5 mm resistor installed on it. Wires with a diameter of 0.51 mm and a length of 45 mm were connected to the resistor using solder. This length was because, in the experiment conducted at this length, the temperature that the wire was comparable to the ambient temperature. In addition, based on the experimental results, the work piece dimensions were reduced since this should not have affected the heat distribution pattern, but would increase the calculation speed.

**Figure 4:**
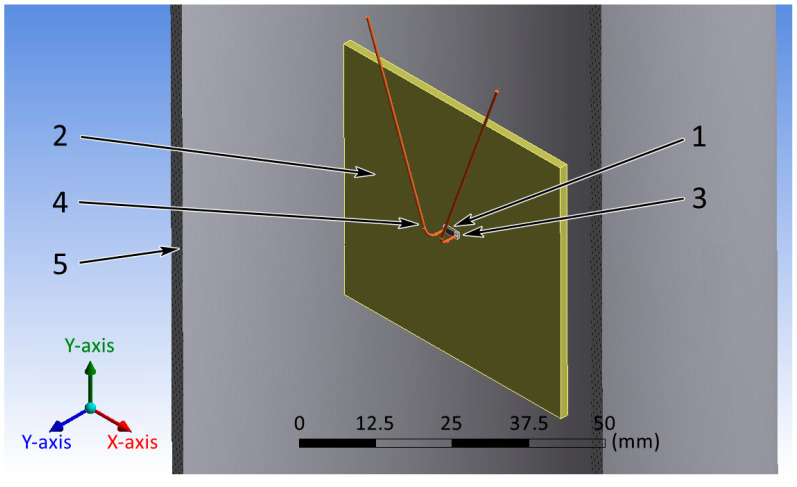
Cross-section of the final view of the geometric model: 1—component; 2—base; 3—solder; 4—wire; 5—cylinder.

To solve the CFD problems, it was necessary that the geometric model had a finite volume. To determine this volume, we placed the 3D model of the work piece in the center of the cylinder (Figure 4). The entire volume inside the cylinder, excluding the volume of the PCB, was filled with air. The cylinder was larger than the researched work piece to exclude the cylinder’s influence on the simulation results: the outer diameter of the cylinder was 105 mm, the inner diameter was 100 mm, the height was 200 mm. Thus, a geometric model had been obtained, which needed to be divided into finite elements.

**Figure 5:**
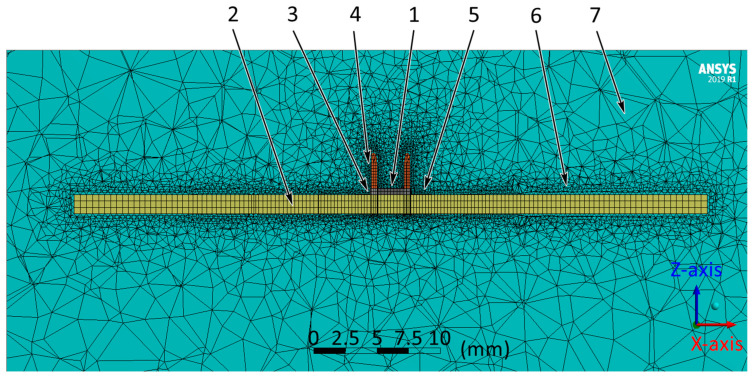
The cross-section of the grid created on the geometric model: 1—component; 2—base; 3—solder; 4—wire; 5—the air around the component, solder and wires; 6—the air around the base; 7—air.

In the model, the part of the airflow located next to the component and the base made of fiberglass was of the most significant interest since it carries out the convective heat transfer from the component. Therefore, the linear dimensions of the mesh in the component and base areas were reduced to 0.25 mm and 0.5 mm, respectively. As a result, the following grid was created on the geometric model (Figure 5). Below are the specified shape and the maximum allowable linear size of the mesh element for each model object (Table 1).

**Figure 6:**
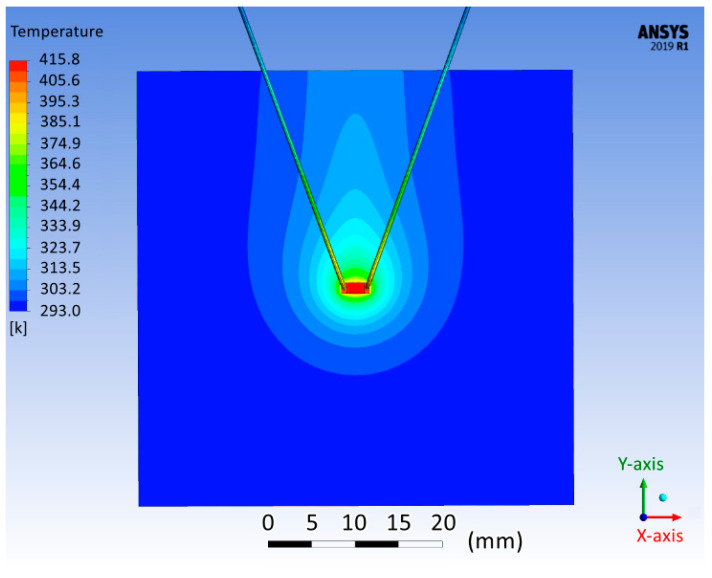
Heat distribution on the surface of the fiberglass base in the simulation model.

The initial conditions of the process were also set:

1. The acceleration of gravity was opposite to the Y-axis (Figure 5 and Figure 6) and was modulo $9.81\;\frac{m}{s^{2}}$;
2. The external pressure was $10^{5}\;\mathrm{Pa}$;
3. The air density was set to a constant equal to $1.225\;\frac{\mathrm{kg}}{m^{3}}$.

Next, the properties of solid materials were set, namely the following: density, specific heat capacity, thermal conductivity (Table 2).

It was also necessary to determine the air parameters (Table 3). Since the model did not assume the transition of air from a gaseous state to a liquid state, its behavior could be fully described by the model of an ideal gas.

Further clarification of the conditions of the process were made: during the construction, a standard type of pressure solver was used, as well as an absolute velocity model, since it was assumed that the airflow to the area under study occurred without vortices. The k−ε model simulated the turbulence process [28]. The model was calculated using the SIMPLEC algorithm to calculate the arithmetic mean values of the scalar in adjacent cells separated by a face (green Gauss cell base).

### 2.3. Analysis of the Constructed Simulation Model

When comparing the obtained picture (Figure 6) with the result of the experiment (Figure 3), it could be seen that the results had a similar shape, with minor differences. The component’s temperature in the simulation model was 415.8 K, comparable to the temperature values obtained during the experiment (see Section 2.1).

Thus, the created model with sufficient accuracy reflected the investigated heating processes, which allowed it to be used in further experiments.

## 3. Results

The constructed simulation model gave us the opportunity to study the cooling processes of the PCB under conditions of natural and forced convection. In order to do this, we needed to set the direction of the forced airflow in the model. It could be assumed that the forced movement of air from top to bottom with small values of the airflow velocity would prevent the natural removal of heat from the PCB. However, how much this phenomenon affected the component’s temperature was unknown. Therefore, to investigate this aspect in the model, the forced airflow was directed from top to bottom.

**Figure 7:**
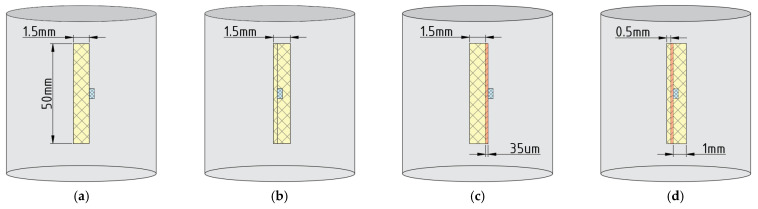
Geometric models of the experiment (side view): (**a**) surface component, without a copper layer; (**b**) embedded component, without a copper layer; (**c**) surface component, with a copper layer; (**d**) embedded component, with a copper layer.

Four models were created based on the previously obtained results to analyze the effect of the airflow velocity on the temperature of the surface and embedded component (Figure 7).

Usually, in real printed circuit boards, the components have a permanent conductive heat sink through the conductors. In future modeling, we will consider only boundary cases. In the first case (Figure 7a,b), the components were attached directly to the fiberglass. In the second case (Figure 7c,d), the components were attached to a copper polygon without any conductive pattern.

Similar features of the models were a 50×50×1.5 mm FR-4 fiberglass base with an attached component 0805 was placed in the cylinder. The component, in turn, was a heat source with a volumetric power density equal to $0.654\;\frac{W}{{\mathrm{mm}}^{3}}$. Again, the initial conditions described above were used (Section 2.1 and Section 2.2).

**Figure 8:**
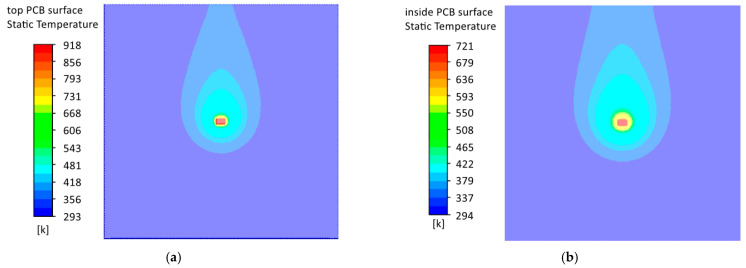
Heat distribution according to models without a copper layer under natural convection conditions: (**a**) surface component; (**b**) embedded component.

**Figure 9:**
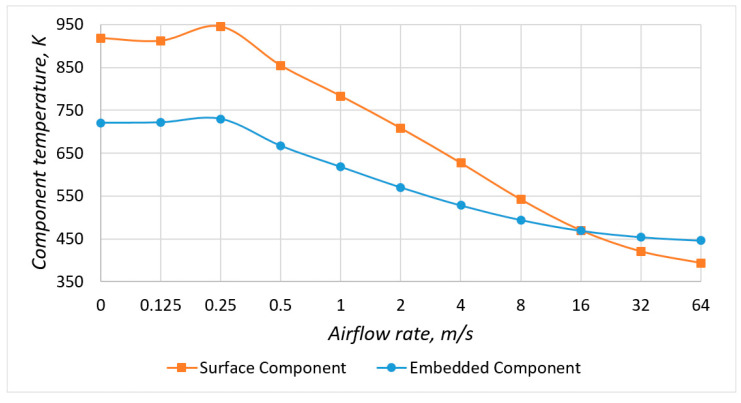
Dependence of the component temperature on the airflow rate in models without a copper layer.

Based on the simulation results (Figure 8), graphs of the component temperature dependence on the input current velocity (Figure 9) were constructed for models without a copper layer and the following conclusions were made:

1. Under conditions of natural and forced convection (at airflow values up to $16\;\frac{m}{s}$), the temperature of the embedded component was lower than the temperature of the surface component. This phenomenon was because the embedded component dissipated its power only into the base material, which increased the dispersion area and cooling efficiency. On the other hand, fiberglass has a low thermal conductivity, which did not allow effective cooling of the built-in component at high airflow rates.
2. The components’ temperature increased at low airflow velocity values, confirming the assumption made earlier. However, it could be noticed that the temperature of the components reached its maximum at a forced flow rate of $0.25\;\frac{m}{s}$. This velocity value could be considered the absolute value of air velocity due to natural convection. In this case, the component temperatures were 11 K and 9 K higher than the natural cooling for the surface and embedded components.

From a practical point of view, the existing equipment cooling systems produced a flow rate of no more than $15\;\frac{m}{s}$. However, in real conditions, the airflow velocity in electronic equipment does not exceed $3\;\frac{m}{s}$. Therefore, the following conclusion could be drawn: in most use cases, namely in conditions of natural cooling and low-performance forced cooling, the built-in component will have a lower temperature compared to the surface one, which would positively affect its reliability and the reliability of the electronic device as a whole.

**Figure 10:**
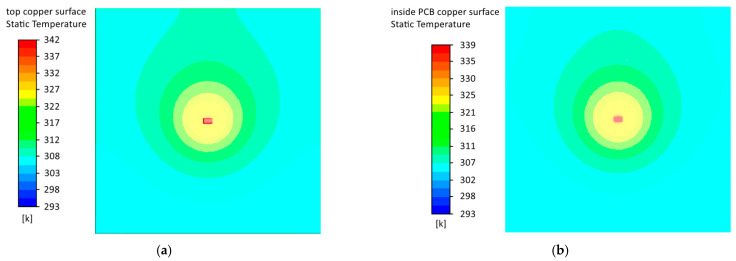
Heat distribution according to models with a copper layer under natural convection conditions: (**a**) surface component; (**b**) embedded component.

**Figure 11:**
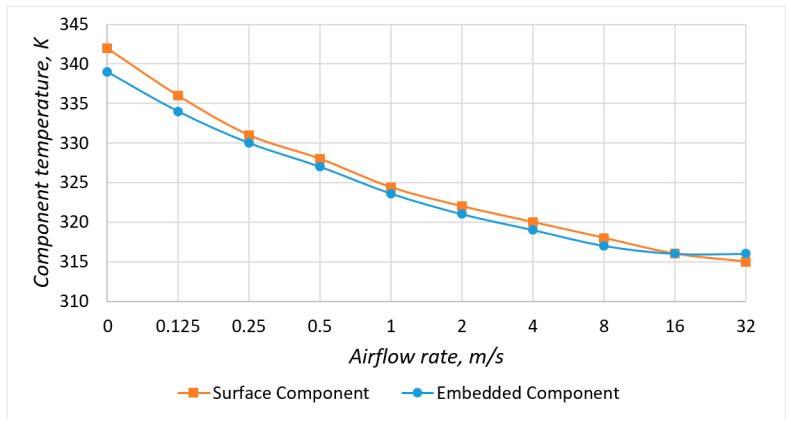
Dependence of the component temperature on the airflow rate in models with a copper layer.

Let us consider models with a copper layer (Figure 10) and graphs of the component temperature dependence on the input flow rate (Figure 11). The introduction of a copper layer significantly reduced the temperature of the component and the temperature gradient on the PCB. When the flow velocity of the blown air was less than $16\;\frac{m}{s}$, the temperature of the embedded component was lower than the temperature of the surface component, which was similar to models without a copper layer. Under natural cooling conditions, the difference between the components’ temperatures was about 3 K, and in the presence of forced cooling was about 1.5 K. However, the number of components on the real PCBs was tens and hundreds, and their power could be higher than in the considered model. In this case, the advantage of the embedded mounting would be more significant.

Also, in models with a copper layer, there was no increase in temperature at a low airflow rate, which indicated that cooling naturally was insignificant in this case due to the slight difference between the temperatures of the PCB and the environment. Supplementary Materials for the article include the results of simulation of heat distribution by models under conditions of forced convection at different airflow rates.

## 4. Conclusions

Currently, the study of the physical reliability of electronic devices and the development of new methods to improve it are significant, especially for thermal processes. For these purposes, we created a simulation model of heat distribution on a printed circuit board, which took into account the processes of conductive and convective heat exchange. The reliability of the model was confirmed by experiment.

Then, based on the constructed model, we investigated the dependence of the component temperature on the cooling method (natural convection and forced convection with variation of the air flow). The results showed that the temperature of the embedded component was less than the temperature of the surface-mounted component under natural convection and, in most cases, of forced convection. The surface component cooled better under conditions of super-efficient forced convection, which are usually not available in real equipment. To do this, the airflow velocity needed to be greater than $16\;\frac{m}{s}$.

The simulation results showed that the temperature of the component, practically, did not depend on the installation method using a copper polygon. However, in modern multilayer printed circuit boards, polygons function as power supply routes. They are located in the middle of the PCB package, but not on its surfaces. In this case the surface component could be connected to the polygons only through the transition holes, which would further worsen its cooling.

It is important to note that the embedded component had the most significant advantage in the absence of forced cooling, which is typical for embedded systems. Thus, the embedded mounting technology reduced the temperature of the component. This phenomenon reduced the thermal effect of the component on the entire electronic device, which increased its overall reliability [29]. However, the question of the thermal efficiency of the integrated mounting with a high density of components on the inner layers of the printed circuit board remains open. In addition, in order to obtain a complete picture of the reliability of PCBs with embedded components, additional studies are necessary using conditions of vibration, humidity and dust.

## Figures and Tables

**Table 1:** Mesh parameters.

Element of Model	Maximum Linear Size of Cell, mm	Shape of Cell
Component	0.25	Prism
Solder	0.25	Prism
Wires	0.25	Prism
Base	0.5	Prism
Cylinder	5	Prism
Air	5	Tetrahedron
Air (around component, solder and wires)	0.25	Tetrahedron
Air (around base)	0.5	Tetrahedron

**Table 2:** Properties of the solid materials used.

Element of Model	Material	Density, $\frac{\mathrm{kg}}{m^{3}}$	Specific Heat Capacity, $\frac{J}{kg\cdot K}$	Thermal Conductivity, $\frac{W}{m\cdot K}$
Base	FR-4 fiberglass	1850	1300	0.343
Solder	Sn63Pb37	1890	250	82
Wires	Copper (Cu)	8900	381	395
Component	$\mathrm{Alumina}\;({\mathrm{Al}}_{2}O_{3})$	3690	880	18

**Table 3:** Air properties.

**Specific Heat Capacity,** $\frac{J}{\mathrm{kg}\cdot K}$	1006.43
**Thermal Conductivity,** $\frac{W}{m\cdot K}$	$2.42\cdot 10^{-2}$
**Viscosity,** $\frac{\mathrm{kg}}{m\cdot s}$	$1.79\cdot 10^{-5}$
**Molecular Weight,** $\frac{\mathrm{kg}}{\mathrm{mol}}$	$2.9\cdot 10^{-2}$

## Data Availability

Not applicable.

## Supplementary Materials

The following supporting information can be downloaded at: https://zenodo.org/record/6301748#.YhwXqehBzct (Accessed 13 April 2022).
